# Death of preceding child and maternal healthcare services utilisation in Nigeria: investigation using lagged logit models

**DOI:** 10.1186/s41043-018-0154-0

**Published:** 2018-11-07

**Authors:** Joshua O. Akinyemi, Izzatullah Bolajoko, Babatunde M. Gbadebo

**Affiliations:** 1 Department of Epidemiology and Medical Statistics, Faculty of Public Health, College of Medicine, University of Ibadan, Ibadan, Oyo State Nigeria; 2 Demography and Population Studies Programme, Schools of Public Health and Social Sciences, University of the Witwatersrand, Johannesburg, South Africa

**Keywords:** Preceding child survival, Childhood mortality, Maternal healthcare, Antenatal care, Skilled attendant at birth, Postnatal care, Lagged logit models, Nigeria

## Abstract

**Background:**

One of the factors responsible for high level of childhood mortality in Nigeria is poor utilization of maternal healthcare (MHC) services. Another important perspective which has been rarely explored is the influence of childhood death on MHC service utilization. In this study, we examined the relationship between death of preceding child and MHC services utilization [antenatal care (ANC), skilled attendant at birth (SAB), and postnatal care (PNC)] among Nigerian women and across the six geo-political zones of the country.

**Methodology:**

We analyzed reproductive history dataset for 16,747 index births extracted from the 2013 Nigeria Demographic and Health Survey. The main explanatory variable was survival status of preceding child; therefore, only second or higher order births were considered. Analysis involved the use of descriptive statistics and lagged logit models fitted for each measure of MHC utilization. Association and statistical significance were expressed as adjusted odds ratio (AOR) with 95% confidence interval.

**Results:**

The use of MCH services for most recent births in the 2013 Nigeria DHS were ANC (56.0%), SAB (34.7%), and PNC (27.3%). Univariate models revealed that the death of preceding child was associated with lesser likelihood of ANC (OR = 0.64, CI 0.57–0.71), SAB (OR = 0.56, CI 0.50–0.63), and PNC (OR = 0.65, CI 0.55–0.69). Following adjustment for maternal socio-economic and bio-demographic variables, statistical significance in the relationship disappeared for the three MHC indicators: ANC (AOR = 1.00, CI 0.88–1.14), SAB (AOR = 0.97, CI 0.81–1.15), and PNC (AOR = 0.95, CI 0.83–1.11). There were no significant variations across the six geo-political regions in Nigeria. The likelihood of ANC utilization was higher when the preceding child died in Northcentral (AOR = 1.19, CI 0.84–1.70), Northeast (AOR = 1.26, CI 0.99–1.59), and South-south (AOR = 1.19, CI 0.72–1.99) regions while the reverse is the case in Southeast (AOR = 0.39, CI 0.23–0.60). For the Southeast, similar result was obtained for ANC, SAB, and PNC.

**Conclusion:**

Death of a preceding child does not predict MHC services use in Nigeria especially when maternal socio-economic characteristics are controlled. Variations across the Northern and Southern regions did not attain statistical significance. Interventions are needed to reverse the pattern such that greater MHC utilization is recorded among women who have experienced child death.

## Background

Maternal and child health indices are key measures of the health and well-being of a nation. Child and maternal death are still common in many resource poor countries in sub-Saharan Africa, Central Asia, Oceania Caucasus, and Southern Asia [1]. To this end, there is now a renewed focus in Sustainable Development Goal (SDG) 3 to end maternal death as well as preventable infant and child death by 2030 [2]. To achieve this, all countries aspire to reduce infant and child mortality to a minimum of 12 deaths per 1000 live births, as well as to reduce maternal death to less than 70 per 100,000 live births [3]. Sub-optimal utilization of maternal healthcare (MHC) services (such as antenatal care, postnatal care, skilled attendant at birth,) in Africa is one of the reasons for poor health outcomes among women of childbearing age [4]. Regular antenatal and postnatal visits during and after pregnancy is important to reduce the risk of maternal/child illness and mortality. It is impossible to separate infant death from maternal morbidity and mortality because maternal well-being to a large extent determines children wellbeing [5–7].

Previously, studies have been conducted to investigate the factors that impact utilization or non-utilization of MHC services. Maternal education has been noted to be the major determinant that positively influences proper utilization of MHC service [7–11]. This is because education has enabled women to make the right decision regarding accessing proper healthcare services. It equipped women with better knowledge which helped them to jettison unhealthy traditional practices thereby protecting both their health and their children’s health. Maternal age, birth order, or previous reproductive histories are also major factors that influence the decision about utilization of MHC services [8, 12]. The extent of investment and use of MHC service tends to reduce with higher birth orders [11]. Some women might think that they are experienced in the art of childbearing, and feel it is not necessary to visit healthcare providers for higher order births.

Long distance and lack of good road networks to health facilities is another major hindrance to accessing and utlising MHC services [13]. Some other factors that have been identified as determinants of MHC services includes delayed administrative processes and treatment, shortage of qualified staff, previous bad experience at a healthcare facilities, lack of essential drugs and supplies, quality and cost of care, inadequate care, ideal family size, and clinical mismanagement [9, 14, 15]. A study carried out in six African countries (Malawi, Kenya, Ghana, Tanzania, Burkina Faso, and Ivory Coast) found out that the odds of delivering subsequent child in a health facility were higher if antenatal care and skilled delivery were utilized for previous child [16].

Child death undoubtedly takes a huge toll on families and the society at large. It is an experience that could affect both the psychology and reproductive behavior of mothers. Some studies have accessed the odds of index child death if the preceding sibling died, as well as how it affects subsequent fertility behavior and choices [17–20]. Occurrence of childhood death may present with either of two consequences: it could either seriously influence the risk of dying or surviving for the next child and/or go on to negatively impact the fertility and health-seeking behavior of women while pregnant and afterward. Child mortality could also influence fertility patterns in the sense that when infant dies, the bereaved parent usually want to have another child to replace the dead one [20]. It could also trigger more birth for women who have not completed their desired family size [19, 20].

An important line of inquiry is the relationship between the death of a preceding child and MHC utilization for subsequent births. A relevant question in this regard is “does the death of a child spur or discourage women from utilisation of MHC services?” Empirical investigations that address this question is important for programs aimed at awareness/advocacy for uptake of MHC services. If women do not perceive their failure to use MHC services as a possible catalyst for childhood morbidity and mortality, it would be difficult to break the cycle of poor MHC utilization and attendant high maternal and childhood mortality. It is expected that the loss of a child should motivate mothers to adopt proactive measures that can prevent a re-occurrence. However, there is virtually no empirical evidence to support or disprove this notion. In this study, we attempt to fill this knowledge gap by showing evidence from Nigeria, the most populous nation in Africa.

Nigeria represents a suitable setting to investigate this hypothesis because of its pronatalism culture and the prevailing high level of fertility and childhood mortality. Data from the 2013 Nigeria DHS showed that averagely, women give birth to about 5.6 children in their reproductive life [21]. In contrast, some progress has been made in child survival, though not substantial enough to meet targets for developmental goals [22]. Nigeria Government and several international partners have made concerted efforts to improve maternal and child health indices, but the output has not been as desired [23]. Some of these interventions have been implemented in selected states while others were driven nationally. One example is the Midwifery Service Scheme (MSS) which aimed to provide trained midwives for critical areas where health personnel are grossly inadequate. Recent assessment showed that the scheme made very little impact on ANC and SAB [24]. The implication of this is that sustained multiple strategies are needed to achieve desired progress in MHC in Nigeria.

There is an avalanche of published research studies on MHC utilization both at local [9, 25–27] and national scales in Nigeria [10, 11, 28, 29]. The determinants often identified in many of these past studies are socio-economic variables such as education and household wealth index. Few other studies showed that there are socio-cultural barriers such as lack of permission by partners, poor perception of need for MHC, non-satisfaction with quality of services, and poor infrastructure as well as negative attitudes of healthcare workers [14, 30, 31]. Though, these studies provide very useful information for design of intervention programs, it is equally important to understand whether the experience of child loss have any impact on women’s perception about their need for MHC services and eventual utilization. Therefore, in this study, we investigate whether women who have lost a preceding child respond to this bitter experience by properly utilizing MHC services for subsequent births or not. We also assessed if the relationship differ across the six geo-political regions in Nigeria.

## Methods

### Study setting

Nigeria is situated on the west coast of Africa between latitudes 40° and 14° north and longitude 4° and 14° east. It spread across about 923,768 km^2^ of land stretching from the Gulf of Guinea on the Atlantic coast in the south to the fringes of the Sahara Desert in the north. It is bounded in the South by the Atlantic Ocean, in the west by Benin republic in the north by Niger and in the east by Cameroon. Nigeria is the most populous country in Africa and the 14th largest in land mass. The country’s current population is estimated to be 183, 234, 791 [24]. Nigeria has about 374 identifiable ethnic groups. The country is made up of 36 states and a Federal Capital Territory, grouped into six geopolitical zones: Northcentral, Northeast, Northwest, Southeast, South-south, and Southwest. Nigeria has a total fertility rate of 5.5 children per woman [21] with a life expectancy at birth of 53 years and 56 years for male and female respectively [32].

### Data source and variables

This is a retrospective analysis of data from the Nigerian Demographic and Health Survey (NDHS) which was jointly conducted by the National Population Commission and ICF International between February and May 2013. The NDHS is a cross-sectional household survey conducted on representative samples selected across the 36 states in Nigeria [21]. Participants selection was done via a three-stage cluster sampling technique using enumeration areas from 2006 population and housing census as the primary sampling unit. Birth recode data of the NDHS 2013 was used in this study.

The outcome variable for this study was utilization of MHC services (antenatal visits (ANC) and skilled attendant at birth (SAB) and postnatal care (PNC). Data collection and derivation of these variables have been described in a previous publication [31]. Study participants were deemed to have received the three services if the care provider was a doctor, nurse, or midwife.

The main independent variable was survival status of preceding child categorized as dead or alive. We adjusted for other variables which literature [8, 28, 29] has shown to be associated with MHC utilization. The control variables were classified into two. First are maternal socio-economic variables (education, occupation, household wealth index, place of residence, and geo-political region). The second group of control variables represents bio-demographic characteristics (maternal age at child’s birth, marital status, birth order, and birth interval). This study focused on survival of preceding child; therefore, analysis was restricted to women with second or higher order births because women with first order births do not have preceding child. The main analytical sample was 16,747 index children in the birth history data of the 2013 NDHS. Index children are the most recent births. Apart from data on SAB which was collected for all children under age 5 years, ANC and PNC were collected for only for most recent child.

### Analysis and modeling procedure

Descriptive analysis was first carried out to assess the association between explanatory variables and the outcome variables. Results were presented using cross tabulations with percentages. Subsequently, four lagged logit models were fitted to explore the research questions. Logit models were used because the outcome variables had two categories. The lag function included in the model was used to derive the survival status of the immediate preceding child. It was also used to account for the correlation in characteristics shared by successive births to same woman. Model I considered the effect of preceding child survival status; model II controlled for maternal-socio-economic variables and model III adjusted for bio-demographic variables. The fourth was a full model in which the independent effect of survival status of preceding child was ascertained by controlling for all other explanatory variables.

The lagged logit models were of the form

- Model I: *y*_*i*_ = *γ*_0_ + *γ*_1_*B*_*i* − 1_ + *ε*_*i*._
- Model II: *y*_*i*_ = *γ*_0_ + *γ*_1_*B*_*i* − 1_ + *γ*_2_*C*_*i*_ + *ε*_*i*_
- Model III: *y*_*i*_ = *γ*_0_ + *γ*_1_*B*_*i* − 1_ + *γ*_3_*D*_*i*_ + *ε*_*i*_
- Model IV: *y*_*i*_ = *γ*_0_ + *γ*_1_*B*_*i* − 1_ + *γ*_2_*C*_*i*_ + *γ*_3_*D*_*i*_ + *ε*_*i*_

Where ${y}_i=\log \left(\frac{p_i}{1-{p}_i}\right)$*p*_*i*_ is the probability of MHC service utilization for *i*th birth. *γ*_0_ is the intercept while *γ*_1_, *γ*_2_, and *γ*_3_ represent the coefficients for preceding child death, maternal socio-economic factors, and bio-demographic variables respectively. *B*_*i−1*_ is the lag term, and it represents the survival status of the immediate preceding child. *C*_*i*_ and *D*_*i*_ were maternal socio-economic and bio-demographic variables respectively. *ε*_*i*_is the error term which was assumed to follow the binomial distribution.

For all analyses, sampling weight was used to account for the cluster sampling design implemented while collecting data. This was achieved by using the “svy” prefix and estimation of robust standard errors during model fitting in Stata MP version 14. Adjusted odds ratio (AOR) and their 95% confidence were based on robust standard errors which further helped to adjust for the complex sample design of the NDHS 2013.

## Results

### Background characteristics

The background characteristics of the recent births analyzed in this study are presented in Table 1. About half (50.7%) of the births were to mothers with no formal education while 23.7% and 5.3% had secondary and higher education respectively. Maternal employment profile showed that 26.8% were not working and 42.2% were involved in sales. Household wealth distribution also revealed that 24.1% and 16.5% of births were from poorest and richest quintile respectively. Much of the births (65.2%) were domiciled in rural areas. Regional distribution showed that the Northwest (37.8%) recorded the largest percentage while the smallest was from the Southeast (8.0%).

**Table 1:** Background characteristics and maternal healthcare services utilization for most recent births, Nigeria, 2013

Variables	All	ANC	SAB	PNC
	*n* = 16,747			
Maternal socio-economic variables				
Education				
None	50.7	30.5	16.3	23.3
Primary	20.3	24.8	24.3	23.8
Secondary	23.7	35.7	45.6	40.5
Higher	5.3	9.0	13.8	12.4
Occupation				
Not working	26.8	21.2	18.1	18.5
Professional/services	7.8	11.7	15.7	13.7
Sales	42.2	44.2	45.3	47.7
Agriculture/manual	23.3	23.0	21.0	20.0
Household wealth index				
Poorest	24.1	9.5	3.3	7.1
Poorer	22.7	16.3	9.9	13.2
Middle	18.9	21.3	19.2	17.7
Richer	17.8	26.0	28.9	27.1
Richest	16.5	26.9	38.7	34.9
Type of residence				
Urban	34.8	50.8	61.9	56.2
Rural	65.2	49.2	38.1	43.8
Geo-political region				
Northcentral	14.1	16.2	17.8	13.5
Northeast	17.2	12.9	8.2	13.7
Northwest	37.8	26.2	13.2	21.2
Southeast	8.0	12.5	17.1	9.4
South-south	8.9	10.9	13.0	12.0
Southwest	14.1	21.8	30.7	30.2
Bio-demographic variables				
Maternal age at child’s birth				
< 20 years	4.3	2.8	1.8	2.9
20–34 years	70.9	71.1	71.4	72.0
> = 35 years	24.8	26.1	26.8	25.1
Marital status				
Not currently married	3.4	3.9	3.9	4.1
Currently married	96.6	96.1	96.1	96.0
Child’s birth order				
2	19.8	21.8	24.3	23.6
3	18.2	19.4	20.9	20.8
4+	62.0	58.9	54.8	55.6
Child’s birth interval				
< 24 months	19.2	18.7	18.6	18.7
24–36 months	40.2	38.7	37.6	38.6
> 36 months	40.6	42.6	43.8	42.7
Survival status of preceding birth				
Dead	12.8	10.5	8.9	9.6
Alive	87.2	89.5	91.1	90.5

The age pattern at child’s birth followed expected pattern, with 70.9% of babies being born to women aged 20–34 years and most of them currently married (96.6%). Births of order 4 (62.0%) and above were quite predominant while 40.6% had preceding birth interval above 36 months. In the total sample, death of preceding child was reported for 12.8%.

### Maternal healthcare services utilization

The use of MCH services for most recent births was ANC (56.0%), SAB (34.7%), and PNC (27.3%). Background characteristics for babies whose mothers utilized these services are also summarized in Table 1. For ANC, about one-third (30.5%) had no formal education while 35.7% attained secondary. Their occupation profile was like that of the total sample. Household wealth distribution of ANC users showed an increasing trend from poorest (9.5%) to richest (26.9%). Also, in contrast to the overall sample, 50.8% of ANC users were resident in urban areas. Distribution for other variables was very similar to that already described for the total sample.

For babies who benefited from skilled assistant at delivery, 45.6% had mothers with secondary education while 62% were resident in urban areas (Table 1). Across the six geopolitical regions, the Southwest region (30.7%) constituted the largest percentage among babies whose birth was assisted by skilled personnel. Virtually, all the other variables were similarly distributed as previously described for ANC.

Background profile of PNC users are also presented in Table 1 which showed that 40.5% and 12.4% had secondary and higher education respectively. All the other variables followed similar patterns as reported for skilled assistance at birth.

### Death of preceding child and ANC utilization

Results from models investigating the relationship between death of preceding child and utilization of ANC are summarized in Table 2. Model I revealed that the odds of ANC utilization was 36% lesser for births whose predecessors died (OR = 0.64, CI 0.57–0.71). Following adjustment for maternal socio-economic variables in model II, the statistical significance of the effect of preceding child death on ANC utilization disappeared (AOR = 0.98, CI 0.87–1.12). Model III in which only bio-demographic variables were controlled showed results similar to model I. The death of a preceding child was significantly associated with lower likelihood of ANC utilization (AOR = 0.67, CI 0.61–0.75). From the full model (model IV), there was no relationship between death of preceding child and use of ANC (AOR = 1.00, CI 0.88–1.14). Other maternal socio-economic variables found significantly associated include education, occupation, household wealth index, type of residence, and geo-political region. The likelihood of ANC utilization increased with educational attainment and household wealth index. Similarly, mothers engaged in professional/services employment (AOR = 1.50, CI 1.18–1.90) were more likely than the unemployed to have used ANC services. Babies born in rural areas were also less likely to have received ANC during pregnancy (AOR = 0.59, CI 0.52–0.67). Significant regional differentials were observed in Northeast (OR = 0.66, CI 0.54–0.81), Northwest (AOR = 0.57, CI 0.49–0.69), and South-south (AOR = 0.39, CI 0.32–0.48) compared to the Southwest.

**Table 2:** Relationship between preceding child death, socio-economic, bio-demographic variables, and antenatal care utilization, Nigeria, 2013

Variables	Model I	Model II	Model III	Model IV
	OR (95% CI)	AOR (95% CI)	AOR (95% CI)	AOR (95% CI)
Maternal socio-economic variables				
Education				
None		1.00		1.00
Primary		2.17(1.93–2.44)*		2.18(1.94–2.45)*
Secondary		3.28(2.83–3.79)*		3.37(2.92–3.91)*
Higher		7.22(4.85–10.75)*		7.29(4.89–10.86)*
Occupation				
Not working		1.00		1.00
Professional/services		1.52(1.20–1.93)*		1.50(1.18–1.90)*
Sales		1.29(1.16–1.44)*		1.25(1.12–1.40)*
Agriculture/manual		1.28(1.14–1.45)*		1.24(1.10–1.41)*
Household wealth index				
Poorest		1.00		1.00
Poorer		1.82(1.62–2.05)*		1.83(1.63–2.06)*
Middle		3.25(2.85–3.72)*		3.26(2.85–3.72)*
Richer		5.72(4.84–6.76)*		5.74(4.85–6.78)*
Richest		7.66(6.01–9.75)*		7.68(6.04–9.78)*
Type of residence				
Urban		1.00		1.00
Rural		0.58(0.52–0.66)*		0.59(0.52–0.67)*
Geo-political region				
Northcentral		0.83(0.68–1.02)		0.84(0.69–1.03)
North east		0.65(0.54–0.80)*		0.66(0.54–0.81)*
Northwest		0.57(0.47–0.69)*		0.57(0.47–0.69)*
Southeast		1.00(0.77–1.31)		0.99(0.76–1.30)
South-south		0.39(0.32–0.48)*		0.39(0.32–0.48)*
Southwest		1.00		1.00
Bio-demographic variables				
Maternal age at child’s birth				
< 20 years			1.00	1.00
20–34 years			2.93(2.40–3.57)*	1.13(0.90–1.43)
> = 35 years			3.86(3.11–4.79)*	1.31(1.02–1.68)*
Marital status				
Not currently married			1.00	1.00
Currently married			0.69(0.57–0.85)*	0.95(0.75–1.20)
Child’s birth order				
2			1.00	1.00
3			0.80(0.70–0.90)*	0.98(0.84–1.15)
4+			0.54(0.49–0.60)*	1.03(0.90–1.18)
Child’s birth interval				
< 24 months			1.00	1.00
24–36 months			0.91(0.82–1.00)	1.02(0.91–1.15)
> 36 months			1.05(0.95–1.17)	1.14(1.01–1.29)*
Survival status of preceding birth				
Dead	0.64(0.57–0.71)*	0.98 (0.87–1.12)	0.67(0.61–0.75)*	1.00(0.88–1.14)
Alive	1.00	1.00	1.00	1.00
Model parameters				
Log likelihood	− 11,422.43	− 8404.44	−11,243.46	− 8389.31
*p* value (Wald statistic)	< 0.001	< 0.001	< 0.001	< 0.001

* (*p* <0.05)

### Death of preceding child and skilled assistance at birth

Table 3 presents the relationship between preceding child death and skilled assistance at next birth among Nigerian women. In model I which include only the survival status of preceding child, the odds of SAB was lower when the preceding child is dead (OR = 0.56, CI 0.50–0.63). Addition of maternal socio-economic variables (model II) led to the disappearance of significant association found in model I (AOR = 0.95, CI 0.80–1.13). Model III which adjust for only bio-demographic variables showed that apart from death of preceding child (AOR = 0.60, CI 0.53–0.68), maternal age at child’s birth, marital status, child’s birth order, and birth interval was also significantly associated with SAB. From model IV, preceding child death was not associated with SAB (AOR = 0.97, CI 0.81–1.15) while most of the maternal socio-economic variables exhibited similar relationship as reported for ANC. Specifically, use of SAB increased with levels of education and household wealth index. Similarly, SAB was less likely in rural than urban areas (AOR = 0.62, CI 0.55–0.69); Northcentral, Northeast, Northwest, and South-south regions compared to Southwest.

**Table 3:** Relationship between preceding child death, socio-economic, bio-demographic variables, and skilled assistance at birth, Nigeria, 2013

Variables	Model I	Model II	Model III	Model IV
	OR (95% CI)	AOR (95% CI)	AOR (95% CI)	AOR (95% CI)
Maternal socio-economic variables				
Education				
None		1.00		1.00
Primary		1.84(1.60–2.10)*		1.84(1.61–2.11)*
Secondary		3.06(2.64–3.56)*		3.16(2.71–3.67)*
Higher		9.06(6.58–12.48)*		9.09(6.58–12.55)*
Occupation				
Not working		1.00		1.00
Professional/services		1.18(0.94–1.49)		1.16(0.92–1.46)
Sales		1.17(1.02–1.33)*		1.13(0.98–1.29)
Agriculture/manual		1.06(0.91–1.23)		1.03(0.88–1.19)
Household wealth index				
Poorest		1.00		1.00
Poorer		2.29(1.88–2.79)*		2.29(1.88–2.79)*
Middle		4.13(3.38–5.04)*		4.11(3.36–5.01)*
Richer		6.08(4.91–7.53)*		6.06(4.89–7.50)*
Richest		11.06(8.59–14.25)*		10.98(8.52–14.14)*
Type of residence				
Urban		1.00		1.00
Rural		0.61(0.54–0.69)*		0.62(0.55–0.69)*
Geo-political region				
Northcentral		0.75(0.62–0.89)*		0.75(0.63–0.90)*
Northeast		0.29(0.24–0.35)*		0.29(0.24–0.35)*
Northwest		0.20(0.16–0.23)*		0.20(0.16–0.24)*
Southeast		1.27(1.02–1.57)*		1.26(1.02–1.57)*
South-south		0.41(0.34–0.49)*		0.40(0.33–0.49)*
Southwest		1.00		1.00
Bio-demographic variables				
Maternal age at child’s birth				
< 20 years			1.00	1.00
20–34 years			4.74(3.64–6.17)*	1.30(0.96–1.77)
> = 35 years			6.85(5.19–9.05)*	1.56(1.12–2.18)*
Marital status				
Not currently married			1.00	1.00
Currently married			0.79(0.65–0.97)*	1.04(0.81–1.32)
Child’s birth order				
2			1.00	1.00
3			0.75(0.66–0.85)*	0.92(0.78–1.09)
4+			0.43(0.38–0.48)*	0.97(0.84–1.13)
Child’s birth interval				
< 24 months			1.00	1.00
24–36 months			0.88(0.79–0.98)*	0.99(0.86–1.14)
> 36 months			1.03(0.92–1.14)	1.12(0.97–1.29)
Survival status of preceding birth				
Dead	0.56(0.50–0.63)*	0.95(0.80–1.13)	0.60(0.53–0.68)*	0.97(0.81–1.15)
Alive	1.00	1.00	1.00	1.00
Model parameters				
Log likelihood	−10,739.6	− 6745.35	−10,453.92	− 6729.49
*p* value (Wald statistic)	< 0.001	< 0.001	< 0.001	< 0.001

* (*p* <0.05)

### Death of preceding child and use of PNC services

Like ANC and SAB, model I (Table 4) showed that PNC was less likely when the preceding child died (OR = 0.65, CI 0.57–0.74). Subsequent addition of maternal socio-economic variables (model II) indicates that death of preceding child was not significantly associated with PNC services utilization by Nigeria women. Controlling for bio-demographic variables makes little or no impact on the results earlier described in model I (AOR = 0.69, CI 0.60–0.78). From model IV in which all variables were controlled, it was found that preceding child death was not related to PNC utilization (AOR = 0.95, CI 0.83–1.11). Again, maternal socio-economic variables have significant relationship with PNC services utilization.

**Table 4:** Relationship between preceding child death, socio-economic, bio-demographic variables, and postnatal care utilization, Nigeria, 2013

Variables	Model I	Model II	Model III	Model IV
	OR (95% CI)	AOR (95% CI)	AOR (95% CI)	AOR (95% CI)
Maternal socio-economic variables				
Education				
None		1.00		1.00
Primary		1.93(1.69–2.20)*		1.92(1.68–2.20)*
Secondary		2.58(2.23–2.98)*		2.57(2.22–2.97)*
Higher		4.11(3.28–5.16)*		4.10(3.26–5.14)*
Occupation				
Not working		1.00		1.00
Professional/services		1.24(1.03–1.49)*		1.24(1.03–1.50)*
Sales		1.47(1.30–1.66)*		1.47(1.30–1.66)*
Agriculture/manual		1.20(1.05–1.37)*		1.20(1.05–1.38)*
Household wealth index				
Poorest		1.00		1.00
Poorer		1.79(1.51–2.11)*		1.78(1.51–2.10)*
Middle		2.54(2.14–3.02)*		2.53(2.13–3.01)*
Richer		3.71(3.08–4.47)*		3.72(3.09–4.49)*
Richest		4.75(3.83–5.90)*		4.79(3.85–5.95)*
Type of residence				
Urban		1.00		1.00
Rural		0.86(0.76–0.96)*		0.86(0.77–0.96)*
Geo-political region				
Northcentral		0.52(0.45–0.61)*		0.52(0.45–0.61)*
Northeast		0.75(0.66–0.93)*		0.75(0.66–0.93)*
Northwest		0.48(0.41–0.57)*		0.48(0.41–0.57)*
Southeast		0.39(0.32–0.47)*		0.39(0.32–0.47)*
South-south		0.49(0.42–0.58)*		0.49(0.41–0.58)*
Southwest		1.00		1.00
Bio-demographic variables				
Maternal age at child’s birth				
< 20 years			1.00	1.00
20–34 years			2.20(1.72–2.82)*	0.92(0.71–1.20)
> = 35 years			2.61(2.00–3.41)*	0.94(0.71–1.26)
Marital status				
Not currently married			1.00	1.00
Currently married			0.76(0.62–0.94)*	0.80(0.64–1.00)
Child’s birth order				
2			1.00	1.00
3			0.86(0.75–0.98)*	1.00(0.87–1.16)
4+			0.57(0.51–0.64)*	0.99(0.87–1.12)
Child’s birth interval				
< 24 months			1.00	1.00
24–36 months			0.93(0.83–1.05)	1.00(0.88–1.13)
> 36 months			1.03(0.92–1.16)	1.00(0.88–1.14)
Survival status of preceding birth				
Dead	0.65(0.57–0.74)*	0.96(0.83–1.11)	0.69(0.60–0.78)*	0.95(0.83–1.11)
Alive	1.00	1.00	1.00	1.00
Model parameters				
Log likelihood	− 9750.86	− 8119.01	− 9647.72	− 8116.12
*p* value (Wald statistic)	< 0.001	< 0.001	< 0.001	< 0.001

* (*p* <0.05)

### Regional variations

Relationship between death of preceding child and utilization of the three MHC services across the six geo-political regions in Nigeria is summarized in Table 5. Pattern of the confidence intervals (CIs) for the AOR was used for indirect assessment of variation across regions. Specifically, overlapping CIs implied no significant difference between regions. The results showed that there are very minimal regional differences. The likelihood of ANC utilization was higher when the preceding child died in Northcentral (OR = 1.19, CI 0.84–1.70), Northeast (AOR = 1.26, CI 0.99–1.59), and South-south (AOR = 1.19, CI 0.72–1.99) regions while the reverse is the case in Northwest and Southeast. Statistical significance was attained only in Southeast (AOR = 0.39, CI 0.23–0.66). That is, only the Southeast region differs from the other five which are all similar.

**Table 5:** Relationship between preceding child death and MCH services utilization across geopolitical regions, Nigeria 2013

Geo-political region	ANC	SAB	PNC
	AOR (95% CI)	AOR (95% CI)	AOR (95% CI)
Northcentral	1.19 (0.84–1.70)	1.30 (0.90–1.88)	1.38 (0.93–2.07)
Northeast	1.26 (0.99–1.59)*	0.90 (0.62–1.32)	1.44 (1.09–1.91)**
Northwest	0.99 (0.82–1.19)	1.20 (0.87–1.65)	0.77 (0.58–1.01)*
Southeast	0.39 (0.23–0.66)**	0.62 (0.39–0.97)**	0.64 (0.39–1.06)*
South-south	1.19 (0.72–1.99)	0.87 (0.54–1.42)	0.98 (0.63–1.50)
Southwest	1.02 (0.52–1.99)	0.79 (0.50–1.27)	0.89 (0.59–1.36)

*p < 0.1; **p < 0.05

For SAB, preceding child death was associated with higher odds in Northcentral (AOR = 1.30, CI 0.90–1.88) and Northwest (AOR = 1.20, CI 0.87–1.65). In the other regions, the likelihood of SAB was lower when the preceding child is dead, with statistical significance observed again in the Southeast (AOR = 0.62, CI 0.39–0.97). All the CIs overlapped; therefore, there was no significant variation in the relationship between death of preceding child and SAB across regions.

In the Northcentral and Northeast regions, the likelihood of PNC utilization was higher when the preceding child is dead while the reverse pattern was observed in the other regions. Similar to SAB, there was no significant variation in the relationship between death of preceding child and PNC utilization across the six regions in Nigeria.

## Discussion

This study sought to examine the effects of preceding child death on utilization of ANC, SAB and PNC services using data from the Nigeria Demographic and Health Survey of 2013. Unadjusted models showed that the likelihood of maternal healthcare utilization was lower when a preceding child is dead. However, adjusted models revealed that the relationship was fully explained by maternal socio-economic factors. This implied that maternal socio-economic variables exhibited some confounding effect on the relationship between the main explanatory and the outcome variables. In summary, while bivariate analyses showed that death of preceding child was associated with non-utilization of MHC services, multivariate analyses suggested there was no relationship.

The pattern of bivariate and multivariate results has very serious public health implications. Apparently, child loss did not motivate better utilization of MHC services during subsequent pregnancies by Nigerian women. This might explain why childhood death clustering (concentration of children deaths in few women) is very common especially in Northern Nigeria [33–36]. Aside from other complex factors, this may also explain why the progress in maternal and child health indices has been very slow in the country. Investigations in Northern Nigeria revealed that lack of perceived need is one of the reasons for non-utilization of MHC services [30]. It is surprising that utilization of MHC services is poor in a setting where fertility and childhood mortality is very high. This could be so because the people did not see a link between non-utilization of MHC services and risk of childhood deaths. This is also in tandem with findings from a previous study that revealed poor awareness of dangerous pregnancy signs in Northern Nigeria [37]. Death of a preceding child should be motivate women to take preventive measures, but our results suggest otherwise. Advocacy programs are needed to bring this potential linkage to the attention of women.

The confounding role of socio-economic characteristics is not surprising because previous studies have shown that they are often significantly related to MHC utilization [8, 38] and childhood mortality [22, 39, 40]. For instance, MHC utilization is associated with better education; whereas, the latter plays protective role against childhood mortality. Women who lost preceding child were more likely to have lower education; similarly, they were less likely to utilize MHC services. With this scenario, inclusion of preceding child survival status and education in the same model would result in the former losing statistical significance.

Although no significant variations across most regions, results revealed that in the Northcentral and Northeast, death of preceding child was associated with better MHC utilization. In the Northwest, only SAB was positively affected. In other words, Northcentral and Northeast women tend to avail themselves of MHC services if they have experienced child loss in the past. Although, detailed empirical explanation for this is beyond the scope of the data analyzed in this study, contemporary information from the sub-regions provide a clue. There are some interventions that have been implemented to salvage maternal and child health in the Northern regions [15, 37, 41]. A good example of this is the PRIN-MNCH program with components including community-based advocacy and awareness about childhood morbidity, mortality, and preventive strategies [42]. Women who have lost children in the past might have been sensitized through such interventions such that they were motivated to avail themselves of MHC services. This explanation is not a verdict on the effectiveness of the afore-mentioned interventions but possible ripple effects. Non-significance of the effect of preceding child death on MHC utilization in the Southwest and South-south is not surprising because of the positive disposition to MHC services in these regions [6, 43].

The result for the Southeast region showed that for the three MHC services (ANC, SAB, and PNC), the likelihood of utilization was significantly lower when the preceding child dies. Previous studies have documented some barriers to MHC utilization and these include poor readiness and lack of infrastructure at health facilities, attitude of health workers, and non-satisfaction with quality of services provided [44]. These, coupled with loss of a previous child could discourage women from adequate uptake of MHC. Favorable dispositions to patronage of traditional birth attendants may also be a factor [26]. Further research is recommended to fully understand the peculiar situation in the sub-region.

The main finding and very minimal variations across sub-regions have implications for perceptions about possible drivers of childhood mortality. This study was premised on the expectation that death of a child might sensitize women to adopt several child survival practices especially utilization of MHC services. Results for the entire country suggest otherwise while regional analyses showed some nuances. Perhaps, childhood mortality perceptions differ across the regions. Scanty evidence suggests that there are cultural beliefs in Southern Nigeria that dead children are from the “spirit world” and when they die, it meant they have returned to where they come from [45]. Such cultural perceptions never linked a child’s death to non-utilization of modern healthcare services. It is relatively unknown if such misconceptions are still common or not. In view of this ambiguity, the position of Montgomery (1998) about mortality perception may still be valid: “we do not yet understand how mortality perceptions are formed and whether they are influenced principally by direct experience, observed experience, or by the acquisition of learning in school, from media or from the health sector itself” [46]. Clearly, new research studies are needed to inform health promotion and advocacy on the drivers of childhood death. Further, the findings in this study serve as eye opener to the need for similar inquiries in other sub-Saharan Africa countries where people’s beliefs and experiences about childhood mortality play very strong roles in MHC utilization.

The significance of maternal socio-economic variables as predictors of MHC utilization is well known from previous studies. These informed the latest drive for universal health coverage as a key strategy for many of the health SDGs [47]. Improvement in female education and employment opportunities would promote better MHC utilization. However, educational improvement is a long-term project. Therefore, short-term strategies are needed to reduce the health inequities across socio-economic strata; this is one of the strong motivations for universal health coverage.

This study has some limitations. The data used for this study was from a retrospective survey which might have been affected by memory recall and unwillingness to recount children death as it may be difficult and painful for the mothers. Underreporting of child deaths may confound the results. Secondly, an important factor that should have been adjusted in addition to survival status of the preceding child is whether MHC services were used for the preceding child. Data on pregnancy and postnatal care was collected only for the index child (most recent birth) in the Nigeria 2013 DHS. These limitations do not overshadow the potential research and programmatic benefits derivable from this study.

## Conclusions

Death of a preceding child does not predict MHC services use in Nigeria especially when maternal socio-economic characteristics are controlled. This is a source of concern because it means that women who experienced death of a preceding child made no concerted effort to avoid a repeat. However, there are variations between the Southeast and other regions. Interventions are needed to reverse the pattern such that greater MHC utilization is recorded among women who have experienced child death. Multiple approaches are needed to increase awareness about the benefits of maternal healthcare and ultimately raised its uptake so that SDG 3 can be achieved. Further studies are needed to understand women’s perception about non-utilization of MHC services as potential risk factors for childhood mortality. This is important because of its relevance to demand creation for maternal healthcare services.

## Abbreviations

ANC: Antenatal care
CI: Confidence interval
MDG: Millennium Development Goal
MHC: Maternal healthcare
NDHS: Nigeria Demographic and Health Survey
OR: Odds ratio
PNC: Postnatal care
SAB: Skilled attendant at birth
SDG: Sustainable Development Goal
UHC: Universal health coverage
